# Comparison of Maternal‐Fetal Attachment and Fertility Motivation in Pregnant Women With and Without Experience of Violence: Descriptive and Analytic Study

**DOI:** 10.1002/hsr2.70512

**Published:** 2025-02-24

**Authors:** Mina Abbasi, Sepideh Dinmohammadi, Roghieh Kharaghani, Maryam Azarkish, Arezoo Haseli

**Affiliations:** ^1^ Social Determinants of Health Research Center, Health and Metabolic Diseases Research Institute Zanjan University of Medical Sciences Zanjan Iran; ^2^ School of Nursing and Midwifery, Zanjan University of Medical Sciences Zanjan Iran; ^3^ School of Nursing and Midwifery Zanjan University of Medical Sciences Zanjan Iran; ^4^ Ayatollah Mousavi Hospital, Zanjan University of Medical Sciences Zanjan Iran; ^5^ Family Health and Population Growth Research Center, Health Policy and Promotion Research Institute Kermanshah University of Medical Sciences Kermanshah Iran

**Keywords:** domestic violence, fertility motivations, maternal fetus attachment, pregnancy

## Abstract

**Background and Aims:**

Domestic violence during pregnancy harms the mother‐child bond and can affect fertility decisions. The study aims to Comparison of Maternal‐Fetal Attachment and Fertility Motivation in Pregnant Women with and without Experience of Violence.

**Materials and Methods:**

This descriptive and analytical study was conducted was conducted in 2024. A total of 292 pregnant women were selected through stratified random cluster sampling from primary healthcare of zanjan, Iran, and divided in non‐violent (147) and violent (145) groups. Data collection tools were Demographic, the Conflict Tactics Scale (CTS2), the mother's attachment to Cranley's fetus, and Miller's fertility preferences questionnaire. To analyze the data by SPSS 17, we used Chi‐square test, Pearson's test, correlation coefficient, and logistic regression, with a significance level set at *p*‐value < 0.05.

**Results:**

Most of the women who experienced violence had a second pregnancy or more (116 out of 145). Unwanted pregnancy was almost twice as common in women who had experienced violence as in women who had not (16.1% vs 8.2%). Women who experienced violence were significantly more likely to have fewer children (χ2 = 4.693, *p* = 0.33). As a result of the Binary logistics regression analyses, it was determined that the variables of young age of husband (OR: 1.325; CI:1.379–3.352), law education level of the husbands (OR:1.313; CI:1.090–2.093), gravida ≥ 5 (OR:5.750; CI:1.018–32.464), low desire to have children (OR:1.882; CI: 1.035–3.420), and low Fetal Attachment (OR: 5.423; CI: 1.965–14.966) were statistically significant with violent women.

**Conclusion:**

Domestic violence during pregnancy affects the bond between mother and fetus, as well as fertility choices. Reducing domestic violence can enhance maternal‐fetal attachment and improve fertility rates.

## 1 Introduction

Violence against women is a serious public health issue and a violation of human rights [1]. Domestic violence, perpetrated by a current or former spouse or partner in an intimate relationship, is the prevalent form of violence against women, impacting 35% of women globally and posing a significant public health problem [2]. A national study in the United States reveals that a staggering 61% of women have encountered physical and sexual violence from their partners [3]. The results of a study conducted in Turkey showed that physical violence is reported at 33.66%, sexual violence at 11.08%, emotional violence at 42.72%, economic violence at 28.78%, and controlling behaviors at 78.53% [4]. Any violent behavior towards women in the domestic setting that is related to gender and results in harm or is associated with the potential for physical, sexual, emotional, or psychological harm is considered domestic violence. This behavior may involve threats, complete denial of authority or freedom, and can occur in public or in secret [1]. Verbal and psychological violence can harm social lives and includes nonverbal threats like finger‐shaking and annoying gestures, as well as verbal threats such as shouting and swearing. Research indicates that women are more vulnerable to violence than men [5, 6].

The impact of domestic violence on pregnant women extends beyond their reproductive health, resulting in both fatal and nonfatal health issues for the developing fetus. This is due to the direct consequences of abuse on the woman's body and the physiological stress effects that arise from ongoing or past abusive situations, which can adversely affect fetal growth and development; Among the various repercussions, one can identify vaginal bleeding, urinary tract infections, the occurrence of premature births, low birth weight, postpartum depression, and other significant mental health issues such as stress, anxiety, depression, and suicidal tendencies [7].

The presence of psychological challenges during pregnancy can have extensive repercussions on multiple facets, particularly affecting the emotional relationship between the mother and her fetus, which pertains to the mother's feelings for her child and violence against women during pregnancy can harm the mother's bond with her baby, and unwanted pregnancies can lead to increased conflict between partners [8, 9]. Research indicates that there exists a notable negative correlation between various forms of domestic violence and the bond between mother and fetus [10]. Thus, early detection and prevention of symptoms disrupting the mother‐fetus bond is crucial to prevent child abuse, cognitive and language development issues, and improve parent‐child relationships, as well as increase positive motivations for couples to have more children [11].

Reducing violence is essential, as it can enhance individual well‐being and significantly lower government expenses [12]. In the last three decades, Iran has witnessed a decrease in fertility rates, as evidenced by census findings showing a drop from around 7.7 children per woman in 1966 to 1.6 in 2011, this data indicates that Iran is currently undergoing replacement fertility [13]. In a society where violence is widespread, a decrease in women's willingness to have children will accelerate the aging of the population and increase the burden of dependents, in such families, unintentional fertility may result in intentional abortion or the birth of children with low self‐confidence, who may exhibit more violent behavior in the future [14]. Miller's research indicated that a strong desire for fertility is correlated with a desire for an increased number of children, a higher preferred number of children, and shorter intervals between births [15]. Nevertheless, negative fertility motivations exhibited a clear inverse relationship with the aspiration to have children and the preferred number of offspring [16]. Politicians recognized the issues of population decline and aging and suggested measures to promote youth and grow the population. These policies aim to support larger families and improve children's lives for a better future [17].

Due to the importance of the mentioned topic, the prevention of domestic violence against women, particularly during pregnancy, has emerged as a crucial issue. Mental health has become increasingly significant in the context of mother‐child relationships in recent years. Targeted interventions are essential to support pregnant women in these situations, ultimately improving outcomes for mothers and their babies. Additionally, there is a growing emphasis on identifying and addressing the needs of abused women as a priority in women's health. Hence, this study's objective is to Comparison of Maternal‐Fetal Attachment and Fertility Motivation in Pregnant Women with and without Experience of Violence in 2024.

## 2 Materials and Methods

### 2.1 Study Setting and Participants

The descriptive analytical/comparative type was implemented in the city of Zanjan, Iran, from 1 January to 15 July, 2024. The study was approved by the Institutional Review Board and Ethical Committee of Zanjan University of Medical Sciences, Iran with an ethical code (IR. ZUMS. REC.1403.017). The inclusion criteria included the mother's age between 18 and 35, Iranian citizenship, basic literacy skills, singleton pregnancy, gestational age between 28 and 40 weeks, residency in Zanjan province, monogamous relationship, no prior marriages, no substance addiction for mother and husband, absence of medical conditions (diabetes, hypertension, heart issues), no obstetrical complications (miscarriages, premature births, ectopic pregnancy), no history of psychological problems (depression, psychosis, stress), no recent stressful events leading to separation from spouse in the past 6 months. The exclusion criteria included reluctance to cooperate for any cause and failure to provide complete answers to the questionnaire.

### 2.2 Sample Size Calculation

The size of the sample was determined based on the preliminary study. With a 10% prevalence of the desire to have children in the violent group and a 15% prevalence in the non‐violent group, along with a first error (*α* = 0.0500), a second error (*β* = 0.20), and a test power of 0.8000, 122 individuals are needed in each group. Factoring in a 20% nonparticipation rate, an estimated 146 individuals are required in each group, totaling 292 individuals.

### 2.3 Data Collection

The study utilized a stratified‐cluster random sampling technique, where Zanjan city was first segmented into four areas: North, South, East, and West. The city is home to a total of 15 health centers. From each region, two centers were selected randomly for sampling (out of 8 health centers in total). Subsequently, the researcher visited the health centers and obtained a list of pregnant women between 28 and 40 weeks pregnant from the integrated health care system's records. Considering the necessary sample size and the quantity of potentially eligible women at each center, a selection of these pregnant women was made through purposive sampling. The designated women were contacted through phone calls, briefed on the goals and methodologies of the investigation, and extended an invitation to visit the pertinent health center. During the face‐to‐face meeting, the specifics of the study were comprehensively presented, and the questionnaires were completed via an interview conducted by the researcher.

After obtaining informed consent, the researchers administered the Conflict Tactics Scale (CTS2) to eligible pregnant women. Based on their completion of the CTS2, the women were categorized into violent (*n* = 145) and non‐violent (*n* = 147) groups.

### 2.4 Measurements

In this research, data collection was conducted using various tools, including a demographic‐obstetric checklist, the conflict tactics scale (CTS2), the mother‐fetus attachment questionnaire (Cranley), and the reproductive preferences questionnaire (Miller). The demographic‐midwifery checklist comprises age, gender, ethnicity, occupations of both males and females, and other midwifery‐related details such as characteristics, gestational age, number of pregnancies, and number of abortions.

#### 2.4.1 Conflict Tactics Scales (CTS2)

The scale, formulated by Strauss et al. (1996) [18] encompasses 36 questions covering physical assault (12 questions), psychological aggression (8 questions), and sexual coercion (4 questions). It also delves into areas such as violence resulting in injury, the injury caused by physical assault (6 questions), and the scale of negotiation, agreement, and non‐agreement in couples' conflict resolution (6 questions). The questionnaire featured a Likert scale with eight options, ranging from 0 to 7. Subject areas that pertain to the same domain are combined, and each area is evaluated independently. Any score above zero indicates the presence of violence, with higher scores corresponds to a more significant level of violence. According to studies conducted abroad, the questionnaire's reliability ranges from 0.79% to 0.97% [18, 19]. This research adopted the Persian translation of the questionnaire, which had been used in prior studies [20], In this study, the questionnaire's validity was established using content validity and consulting with five medical science professors. The reliability of the questionnaire has been established by Cronbach's alpha coefficient across different domains, including the negotiation scale (0.794%), physical domain (0.845%), psychological domain (0.756%), sexual domain (0.766%), and the domain of violence injury (0.753%).

#### 2.4.2 The Mother‐Fetal Attachment Questionnaire

This questionnaire was compiled by Cranley [21]. There are 24 statements in this questionnaire, categorized into five subscales within three dimensions. A: The cognitive dimension addresses distinguishing oneself from the fetus (4 questions) and attributing unique characteristics to the fetus (6 questions). B: The emotional dimension explores selflessness or sacrifice (5 questions). C: Behavioral dimension includes interaction with the fetus (4 questions) and acceptance of the mother's role (5 questions). The responses were evaluated using a 5‐point Likert scale. In particular, Question 22 of the questionnaire was scored in reverse. Consequently, the lowest possible score was 24, while the highest attainable score was 120. Cranley documented a Cronbach's alpha score of 0.85 for the whole questionnaire [21]. In Iran, the Cranley Mother‐Fetal Attachment Scale has been proven to be valid, with both content and face validity, and a high level of internal consistency with a Cronbach's alpha of 0.83. Mother‐fetus dependence is divided into three levels: poor (less than 24), moderate (24 to 48), and good (48 to 120) scores [22].

#### 2.4.3 The Fertility Preferences Questionnaire

by Miller (1995) comprises 10 questions [15]. One of the questions in the questionnaire addresses the desire to have children, and it is measured using a numerical rating scale. The desire to become parents is divided into categories using a numerical scale, with low desire falling between zero and three, moderate desire between three and seven, and high desire above eight. The validity and reliability of the fertility preferences questionnaire were confirmed in Miller's (1995) study in America [15]. Reliable sources were consulted to compile the questionnaire's validity under the supervisor's oversight. Subsequently, the questionnaire was distributed to 10 faculty members and professors with expertise in midwifery, reproductive health, and demographics. The questionnaire's reliability was validated through the test‐retest reliability assessment, resulting in a correlation coefficient of 98% [23].

### 2.5 Measures and Variables

The dependent variable of the study was violence in pregnant women which was measured with CTS2. For the dependent variable, a rate of 1 was given for experience of violence by pregnant women, and 0 did not.

As the experience of violence by pregnant women status was a two‐category dependent variable developed with the options “yes” and “no” binary logistic regression analyses were applied.

The independent variables in this study were determined following the literature review. The socio‐demographic and economic factors that can influence the experience of violence in pregnant women were considered as the independent variables, which were women's age (18–25 years and 26–35 years), husband's age (20–30 years, 31–40 years, and > 40 years), education level of the women (≤ diploma, university education), education level of the husbands (≤ diploma, associated and bachelor science, and master of science and/or above), employment status of the women (employed and housekeeper), employment status of the husbands (employed, self‐employed, and unemployed), level Income (first level income: under 200 Doller, second level income: 200–400 Dollar, and third level income: more than 400 Dollar), location (city and village).

The reproductive factors, fetal attachment (standard scale), and fertility motivation (standard scale) that can influence the experience of violence in pregnant women were also considered as the independent variables, which were number of pregnancies (gravida) (1, 2 −4, and 5 and above), type of pregnancy (wanted, unwanted, and unplanned), desire to have children (low, moderate, and high), desired gender (boy, girl and no different), how many children would you like to have? (1 and ≥ 2), and fetal attachment (low, moderate, and high).

location (two categories), type of pregnancy (three categories) desire gender (three categories) variables included in the model are nominal scale variables.

women's age (two categories), husband's age (three categories), education level of the women (two categories), education level of the husbands (three categories), employment status of the women (two categories), employment status of the husbands (three categories), level Income (three categories), the number of pregnancies (gravida) (three categories), desire to have children (three categories), how many children would you like to have? (two categories), and fetal attachment (three categories) variables are ordinal scale variables.

### 2.6 Statistical Analysis

Descriptive and analytical statistics in SPSS 17 (Statistical Package for Social Science) were used to compare maternal‐fetal attachment, fertility motivation, and sociodemographic factors in pregnant women with and without experience of violence. The frequencies and percentages of the pregnant women participating in the study were obtained in accordance with their experience of violence status. Chi‐square independence tests were carried out to analyze the relationship between violence experience status and independent variables. The significance level is set at a *p* value of less than 0.05.

Binary logistic regression analyses were conducted to determine the socio‐demographic and economic factors affecting violence in pregnant women. Basic assumptions were met in logistic regression. For example, the linearity of the dependent variable and independent variables was tested using a scatter plot. The results of the plots indicated a linear relationship. There were no specific errors (all relevant predictor variables were entered and irrelevant predictors were discarded). Also, the independent variables examined did not have multicollinearity, also we examined outliers in the research samples by the Casewise List table in the binomial logistic regression outputs. Five samples had values above 2 in the ZResid column (these cases do not match our model). These samples were removed and we repeated the test again.

## 3 Results

The frequency analysis of the socio‐demographic, economic, fertility preference, and fetal attachment variables and the chi‐square independence test results are presented in Table 1. The study group included 292 pregnant women who had participated in the cross sectional study in Zanjan, Iran carried out between January and July, 2024.

**Table 1:** Frequency analysis and chi‐square independence test results of the factors that can influence violence in pregnant women.

Variables	Violence (Total = 292)		*n* (%)	*χ*2	*p* value
	Yes	No			
Age (years)					
18–25	51 (17.4)	59 (20.2)	110 (37.6)	0.766	0.381
26–35	94 (32.1)	88 (30.1)	182 (62.2)		
Husband's Age (years)					
20–30	44 (15.1)	38 (13)	82 (28.1)	6.379	**0.041**
31–40	75 (25.7)	95 (32.5)	170 (58.2)		
> 40	26 (8.9)	14 (4.8)	40 (13.7)		
Education level of the women					
≤ Diploma	95 (32.5)	106 (36.3)	201 (68.8)	4.027	0.546
University educations	23 (7.8)	31 (10.6)	54 (18.4)		
Education level of the husbands					
≤ Diploma	118 (40.4)	97 (33.2)	215 (73.6)	11.361	**0.003**
AS and BS	23 (7.8)	34 (11.6)	57 (19.5)		
MS or above	4 (1.3)	16 (5.4)	20 (6.7)		
Employment status of the women					
Employed	8 (2.7)	18 (6.1)	26 (8.9)	4.073	**0.044**
Housekeeper	137 (46.9)	129 (44.1)	266 (91.1)		
Employment status of the husbands					
Employed	21 (7.1)	29 (9.9)	50 (17.1)	1.415	0.234
Self‐employed	70 (23.9)	74 (25.3)	144 (49.3)		
Unemployed	54 (18.4)	44 (15.2)	98 (33.6)		
Level Income					
First level income (lowest)	31 (10.6)	26 (8.9)	57 (19.5)	4.194	0.123
Second level income	111 (38)	111 (38)	222 (76.0)		
Third level income (highest)	3 (1)	10 (3.4)	13 (4.4)		
Location					
City	88 (30.1)	93 (31.8)	181 (61.9)	0.206	0.650
Village	57 (19.5)	54 (18.4)	111 (38)		
Gravida					
1	29 (9.9)	74 (25.3)	103 (35.2)	33.853	**< 0.001**
2–4	70 (23.9)	43 (14.7)	113 (38.6)		
> 5	46 (15.7)	30 (10.2)	76 (26)		
Type of pregnancy					
Wanted	92 (31.5)	111 (38)	203 (69.5)	11.216	**0.004**
Unwanted	47 (16.1)	24 (8.2)	71 (24.3)		
Unplanned	6 (2)	12 (4.1)	18 (6.1)		
Desire to have children					
Low	27 (9.2)	12 (4.1)	39 (13.3)	21.590	**0.001**
Moderate	52 (17.8)	29 (9.9)	81 (27.7)		
High	66 (22.6)	106 (36.3)	172 (58.9)		
Desired gender					
Boy	30 (10.2)	18 (6.1)	48 (16.3)	2.734	0.255
Girl	59 (20.2)	62 (21.2)	121 (41.4)		
No different	58 (19.8)	57 (19.5)	115 (39.3)		
How many children would you like to have?					
1	14 (4.7)	5 (1.7)	19 (6.5)	4.693	**0.033**
≥ 2	131 (44.8)	142 (48.6)	273 (93.4)		
Fetal attachment					
Low	1 (0.3)	0 (0)	1 (0.3)	26.233	**< 0.001**
Moderate	34 (11.6)	5 (1.7)	39 (13.3)		
High	108 (37.0)	138 (47.3)	246 (84.2)		

Abbreviations: AS, associated science; BS, bachelor science; MS, master of science.

The highest participation rate of 21.9% was found in the 25–35 age group and their husband's age was 58.2% in the 30–40 group. In other words, violence against pregnant women was higher at both ends of the partner's age spectrum, (younger and older partners). Only 31.2% of the women and 26.4% of their husband were university academic education. 91.1% of the women were Housekeeper. 33.6% of their husband was unemployed. 76% of the women were of the moderate level income and most of them (61.2%) residence in the city.

Most of the women who experienced violence had a second pregnancy or more (116 out of 145). Unwanted pregnancy was almost twice as common in women who had experienced violence as in women who had not (16.1% vs 8.2%). More desire to have many children was lower in women who had experienced violence than another (36.3% vs. 22.6%). If having children, there was no statistically significant difference in desired gender (boy or girl) between the two groups (*p* = 0.255). Women who experienced violence were significantly more likely to have fewer children (*p* = 0.33). High fetal attachment was more common among women who had not experienced violence (47.3% vs 37%).

Binary logistics regression analyses were conducted to determine the factors that influence the violence of the pregnant women (Table 2). The coefficient (β), standard error and confidence interval values for the binary logistic regression analyses results are presented in Table 2. As a result of the analysis, it was determined that the variables of Husband's Age (Young and old age), Education level of the husbands (under diploma), number of pregnancy (Gravida) (≥ 5), Desire to have children (low and moderate desire), and Fetal Attachment (low attachment) were statistically significant.

**Table 2:** Estimated model results associated with the factors influencing violence in pregnant women.

Variables	Binary logistic regression			
	Adjusted OR	Std. error	95% CI	
			Lower	Upper
Husband's age (reference category: > 40)				
20–30	**1.325**	**0.716**	**1.379**	**3.352**
31–40	0.821	0.386	0.299	1.691
Education level of the husbands (reference category: MS and above)				
≤ Diploma	**1.313**	**0.452**	**1.090**	**2.093**
AS & BS	0.678	.282	.327	1.406
Gravida (reference category: 1)				
2−4	1.339	0.931	0.216	8.298
≥ 5	**5.750**	0.883	**1.018**	**32.464**
Type of pregnancy (reference category: Unplanned)				
Wanted	2.199	0.627	0.643	7.521
Unwanted	2.486	0.664	0.676	9.138
Desire to have children (reference category: High)				
Low	**1.882**	0.305	**1.035**	**3.420**
Moderate	**1.342**	0.229	**1.040**	**4.006**
Housekeeper women	0.722	0.531	0.255	2.046
Desired low number of children	0.254	0.654	0.071	0.917
Low fetal attachment	**5.423**	**0.518**	**1.965**	**14.966**

Abbreviations: AS, associated science; BS, bachelor science; MS, master of science.

## 4 Discussion

The study aims to compare maternal‐fetal attachment and Fertility Motivation in Pregnant Women with and without Experience of Violence. The analysis of the demographic results of this study concerning domestic violence in pregnant women indicated that the husband's age (over 40) and the number of pregnancies (≥ 5) can be considered significant factors. In 2020, Doyle and his colleagues conducted a study demonstrating that the link between a spouse's age, the number of pregnancies, and domestic violence against women is complex. Generally, increased age and more pregnancies, particularly under economic and social pressures, can lead to higher rates of domestic violence [24]. Agarwal, et al. (2023) conducted a review study that demonstrated that pregnancy can create psychological stress for women, which, when combined with economic or social issues, may lead to domestic violence. Repeated pregnancies can further intensify this stress and increase the risk of violence in relationships [25]. Also, our findings indicated that the education level of spouses may play a significant role in affecting domestic violence between partners. Generally, women who did not face violence from their husbands tended to have a higher level of education (OR = 0.678) compared to those who experienced abuse. Enhancing education and involvement in the community can improve communication skills among partners and strengthen conflict resolution skills in marriage problems. Effective communication allows couples to openly tackle marital challenges, strengthening their bond and reducing the risk of violence [26]. Research shows that good communication skills enhance intimacy and lower conflict. Increasing education and community involvement can also improve these skills, aiding in conflict resolution. Studies indicate that strong communication capabilities such as listening, emotional control, and the ability to send and receive messages are linked to lower instances of domestic violence [27, 28].

The results of a study conducted in Turkey showed that risk factors for intimate partner violence (IPV) include partners who abuse alcohol, gamble, cheat, or display aggressive behaviors [4]. Additionally, both low education levels of the women and their partners, along with a history of domestic violence, significantly increase the likelihood of IPV, highlighting the need for effective support systems and interventions for at‐risk women [4]. Also, in a study, the results showed that 45.3% of individuals who faced verbal and psychological violence from their partners had partners who were elementary school graduates. Of these partners, 80.6% were employed, 26.4% used alcohol, and 3.8% gambled [6]. Nevertheless, Soltanifar research revealed no significant link between the educational attainment of both genders and the prevalence of domestic violence [9].

Research in Turkey indicates that low‐income women of childbearing age residing in cities, who have experienced violence outside intimate relationships, are at an increased risk for IPV [4]. However, additional studies suggest that women living in rural and underdeveloped areas are at a higher risk of experiencing sexual violence. Elements like lower levels of education and a history of physical, economic, or verbal abuse increase this danger. On the other hand, older individuals and those with partners or spouses who possess higher educational qualifications typically have a lower likelihood of facing sexual violence [26, 29]. Our study found no significant relationship between domestic violence and place of residence in the two groups (*p* = 0.650). Nevertheless, a statistically significant correlation was found between education level of the husbands and the management of violence within two groups (*p* = 0.003).

The findings of our study indicate a statistically significant difference in the mean scores of maternal attachments to the fetus between the two groups. Specifically, the results demonstrate that pregnant women who have experienced domestic violence exhibit a lower level of maternal attachment to their fetus. All forms of violence, such as physical, mental, verbal, sexual violence, and harm to the mother, are encompassed by this statement. Evidence suggests that exposure to violence and the mental condition of the mother during pregnancy are key factors that influence the mother's attachment to the fetus during pregnancy and following childbirth [30, 31]. A research conducted in Japan revealed a direct correlation between physical violence during pregnancy and postpartum depression, potentially resulting in impaired bonding between mother and infant [32]. Additionally, Soltanifar research indicates a correlation between verbal and physical violence, as well as injuries during pregnancy, and the mother's attachment to the infant [9]. A study has advised that sensitive screening for domestic violence should be considered for women who have been injured during pregnancy, as they may experience a decrease in mother‐fetus attachment to prevent adverse pregnancy outcomes related to violence [31].

Following domestic violence, a woman's mindset, a mother's connection to her unborn child, and her dedication to their health can be profoundly influenced by physical and psychological symptoms, anxiety, lack of social support, and insufficient care [33]. The research conducted by Pires de Almeida and colleagues revealed that pregnant mothers who have encountered violence are prone to developing a negative attitude towards their pregnancy and their fetus. This unfavorable attitude often translates to a diminished connection with the fetus, consequently contributing to elevated risks of fetal complications throughout the pregnancy [34].

Our findings also indicated a statistically significant variance in Fertility Motivation and the longing to have children between the two cohorts. The non‐violent group exhibited a higher average fertility motivation (8.18 ± 2.62) compared to the violence‐exposed group. Furthermore, as the severity of the partner's violence increased, the motivation for fertility decreased, subsequently leading to a decline in the desire to have children and the mother's attachment to the fetus. Consistent with our findings, the research conducted by Ustunsoz et al. revealed an inverse correlation between pregnancy and fetal attachment, indicating that exposure to physical and psychological violence during pregnancy adversely impacts the health of both the mother and the fetus [35]. Negative attitudes towards pregnancy and fertility preferences were found to be more prevalent among abused women in a study, as opposed to non‐abused women. The results of a study indicated that abused women demonstrated a stronger tendency towards negative attitudes regarding pregnancy and fertility preferences in comparison to non‐abused women [14]. Findings from a study conducted in Iran reveal a substantial correlation between the number of children, intentional pregnancy planning, spousal consumption of cigarettes, alcohol, and drugs, family interference, patriarchal attitudes of husbands, and domestic violence against pregnant women [36].

The findings from two separate studies were in opposition to the current study, leading to the conclusion that there was no significant correlation between violence and the fetus's attachment to the mother [3, 37]. The difference could be due to the low incidence of violence in the studies referenced, thereby preventing sexual partner violence from influencing the mother‐infant relationship negatively.

## 5 Limitation of Study

The nondisclosure of violence by pregnant women can be influenced by cultural, social, shame, embarrassment, and forgetfulness. The research conducted in this study utilized a cross‐sectional approach, which limits the ability to establish causal relationships between the variables.

### 5.1 Strength of Study

The authors' use of standard tools to evaluate the variables represents the first strength of this work. Furthermore, the second advantage is the execution of a descriptive‐analytical comparative study between two groups, mothers who have experienced violence and those who have not. The third benefit of conducting sampling across all clinics in the city was achieved through the use of stratified‐cluster random sampling, thereby ensuring the reliability of the results.

### 5.2 Suggestions

It is advisable to pursue additional studies on childbearing trends and domestic violence, and to explore ways to enhance the attachment between mother and fetus. Furthermore, it is crucial to provide personnel with training that aligns with the updated goals.

## 6 Conclusion

The findings of this study indicate that a rise in domestic violence correlates with a decrease in a mother's attachment to the fetus, as well as a decrease in women's intention to have children. Consequently, decreasing instances of domestic violence against women may result in women choosing to have children and ultimately lead to higher fertility rates. Therefore, initiatives aimed at reducing domestic violence against women could potentially boost the overall fertility rate within the nation. Therefore, in the establishment of population policies, policymakers should ensure that they adequately incorporate social and cultural considerations regarding childbearing, in conjunction with economic incentives.

## Author Contributions

**Mina Abbasi:** conceptualization, investigation, funding acquisition, writing–original draft, methodology, validation, visualization, writing–review and editing, software, formal analysis, project administration, data curation, supervision, resources. **Sepideh Dinmohammadi:** conceptualization, resources. **Roghieh Kharaghani:** conceptualization, investigation, formal analysis, project administration, validation, methodology. **Maryam Azarkish:** conceptualization, investigation, visualization, methodology, project administration, software. **Arezoo Haseli:** conceptualization, investigation, funding acquisition, methodology, writing–original draft, formal analysis, data curation, validation, resources, supervision.

## Conflicts of Interest

The authors declare no conflicts of interest.

## Ethics Statement

This article has been approved by the ethics committee of Zanjan University of Medical Sciences under the approval number (IR. ZUMS. REC.1403.017). All study procedures were by the protocol of the Regional Ethical Research Committee and the 1964 Declaration of Helsinki. After knowing about the objectives of the study, written consent was obtained from all women. They were informed that their participation was voluntary, confidential, and anonymous and informed of their right to withdraw from the research at any time. (All women provided written informed consent).

## Transparency Statement

All authors have read and approved the final version of the manuscript. The lead author, Arezoo Haseli, certifies that this manuscript is an honest, accurate, and transparent account of the study reported; that no important aspects of the study have been omitted; and that any discrepancies from the study as planned (and registered, if applicable) have been reported. from the study as planned (and, if relevant, reported) have been explained. The corresponding author had full access to all study data and takes complete responsibility for the integrity and accuracy of the data analysis.

## Data Availability

The data that support the findings of this study are available on request from the corresponding author. The data are not publicly available due to privacy or ethical restrictions. Data in support of the findings of this study are available from the corresponding author upon reasonable request.
